# Photobiomodulation Therapy for the Treatment of Chronic Oral Ulcers: A Report of Two Cases

**DOI:** 10.7759/cureus.67545

**Published:** 2024-08-22

**Authors:** Sakshi Batra, Adit Srivastava, Arjun Mahajan, Fouzia Imran

**Affiliations:** 1 Oral Medicine and Radiology, Faculty of Dental Sciences, Institute of Medical Sciences, Banaras Hindu University, Varanasi, IND; 2 Surgical Oncology, Institute of Medical Sciences, Banaras Hindu University, Varanasi, IND; 3 Periodontics and Implantology, Faculty of Dental Sciences, Institute of Medical Sciences, Banaras Hindu University, Varanasi, IND

**Keywords:** low-level laser, low-level light therapy, ­wound healing, photo-biomodulation, nonhealing chronic wound

## Abstract

Oral ulcers are a very frequent complaint of patients reporting to dental professionals, of which traumatic ulcers are the most common They are very painful and troublesome while the patient speaks, masticates, or brushes. Various treatment modalities, such as topical analgesics and topical or systemic antibiotics, are used conventionally. However, long-term non-healing painful conditions and drug resistance have boosted the rapid raising of an alternative wound healing method. In the presented cases, low-dose biophotonics, also called photobiomodulation (PBM) therapy by low-level laser, was used with the aim of alleviating pain and inflammation, modulating the immune response, and promoting wound healing and tissue regeneration.

## Introduction

Ulcers are a major healthcare burden because of their varied etiologies and inconsistent healing patterns. They are defined by localized tissue injury that leads to the loss of the epidermal layer and connective tissue. These lesions can significantly affect a patient’s quality of life, whether they are single or multiple, acute or chronic, and are frequently recurrent [1,2]. In the oral cavity, trauma-induced ulcers are especially common and usually go away a week after the trigger is removed [3]. Drug resistance is a developing concern that calls for the use of new therapeutic approaches, even though traditional medicines like steroids, antibiotics, and analgesics are often beneficial [4,5].

One method that is gaining popularity is low-level laser treatment (LLLT), which uses the heat produced by laser light to accelerate tissue healing [6]. By targeting particular wavelengths, LLLT promotes rapid wound healing by stimulating cellular responses that improve tissue growth and blood flow. The mechanisms and therapeutic uses of LLLT in ulcer management are covered in detail later in this case presentation, which also highlights the technology’s potential as a useful supplementary therapy option [7].

## Case presentation

Case report one

**Figure 1:**
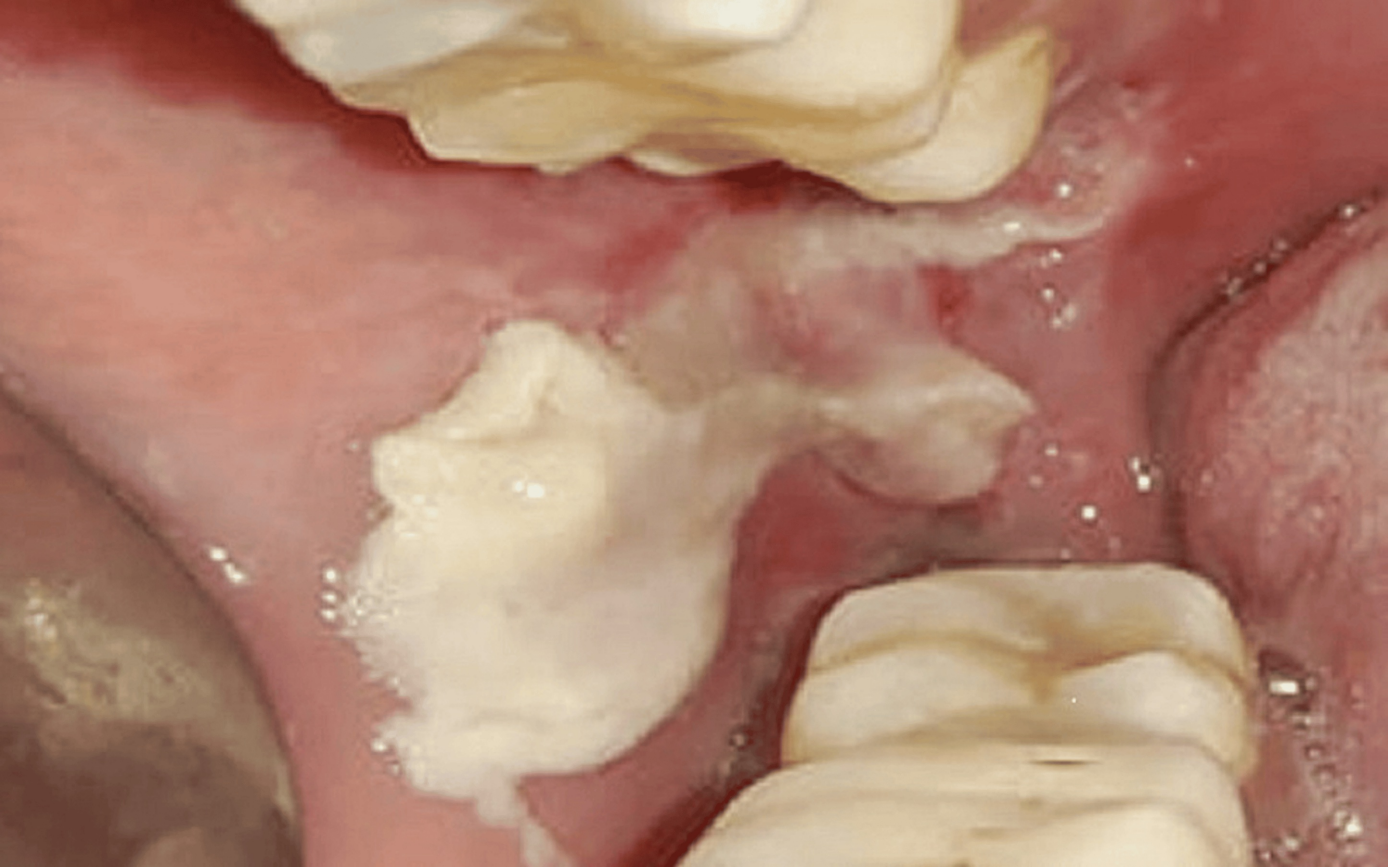
Day one with nonhealing ulcer The initial nonhealing lesion on the right buccal mucosa after trauma during extraction.

A 28-year-old female patient visited the Department of Oral Medicine and Radiology with a chief complaint of pain and an ulcer in the right lower back region after an extraction that occurred 28 days ago. The pain was throbbing and increased while chewing. She had a history of traumatic extraction of the third molar of the right lower posterior region and trauma on the buccal mucosa during the procedure. The patient followed the drug regimen and post-extraction instructions, but the ulcer did not heal and persisted for more than four weeks. On intraoral examination, a well-defined ulcer measuring approximately 2 × 1.5 cm on the right lower third molar region was present. The ulcer extended until the retromolar region and on the buccal mucosa; the margins were erythematous with sloping edges, and the floor was covered with yellowish-white slough (Figure 1).

A radiographic evaluation with an intraoral periapical radiograph showed an extraction socket with slightly resorbed cortical bone. Based on the patient’s history and examination, a provisional diagnosis was made of a nonhealing extraction wound with a traumatic ulcer on the buccal mucosa. The patient was given an explanation for the treatment to be provided, consent was obtained from the patient, and ethical approval was taken for safety.

**Figure 2:**
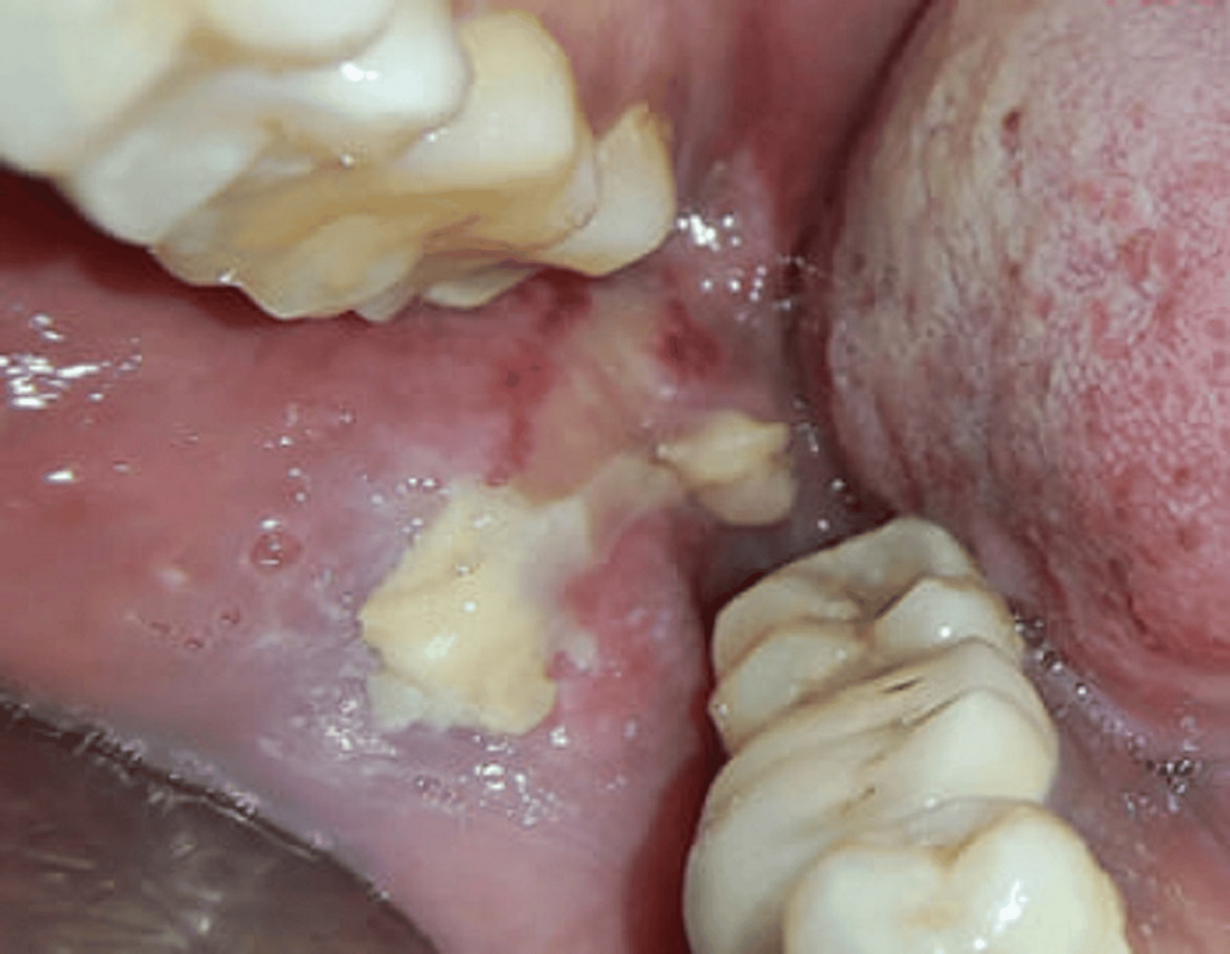
Day two with 10–20% healed lesion The lesion healed 10-20% using a handheld diode laser at 810 nm, 100 mW, 12 J/point, and continuous wave.

The LLLT was administered following all safety measures using a handheld diode laser with 810 nm, 100 mW, 12 J, and continuous wave. Contact was made at five points (one point distal to the socket, one lingual, one buccal to the socket, and two on the buccal mucosa) for two minutes at each point every other day for 10 days. No drugs were used during this procedure. After the first session, the patient’s pain was reduced by 50%, and the wound showed healing of 10-20% (Figure 2).

**Figure 3:**
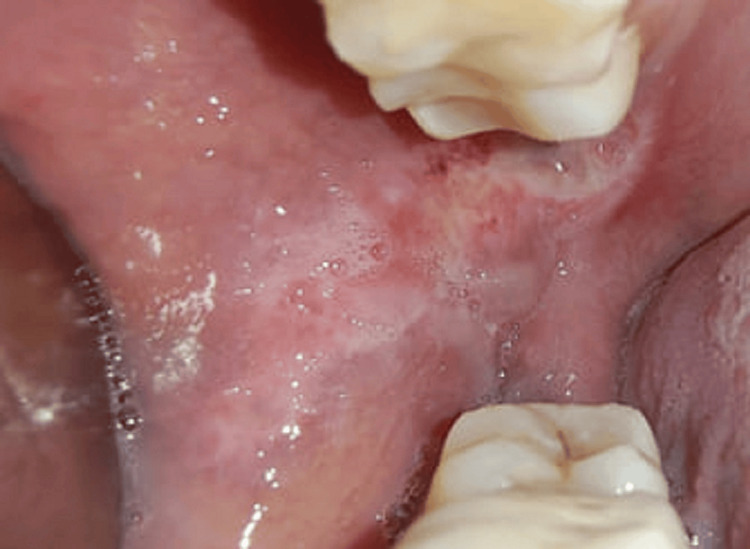
Day 10 with a completely healed ulcer The lesion healed completely on the fifth visit (day 10 of LLLT). The lesion was given LLLT at 810 nm, 100 mW, and 12 J/point. LLLT: low-level laser treatment

On the second visit, LLLT was performed using a handheld diode laser at 810 nm, 100 mW, 12 J/point, and continuous wave. Contact was made for two minutes at each point, and the lesion showed 10-20% healing. By the third visit, there was a 90% reduction in pain and a decrease in the ulcer’s size and inflammation by about 70%. At the fifth visit, no pain was present with satisfactory healing (Figure 3).

Case report two

**Figure 4:**
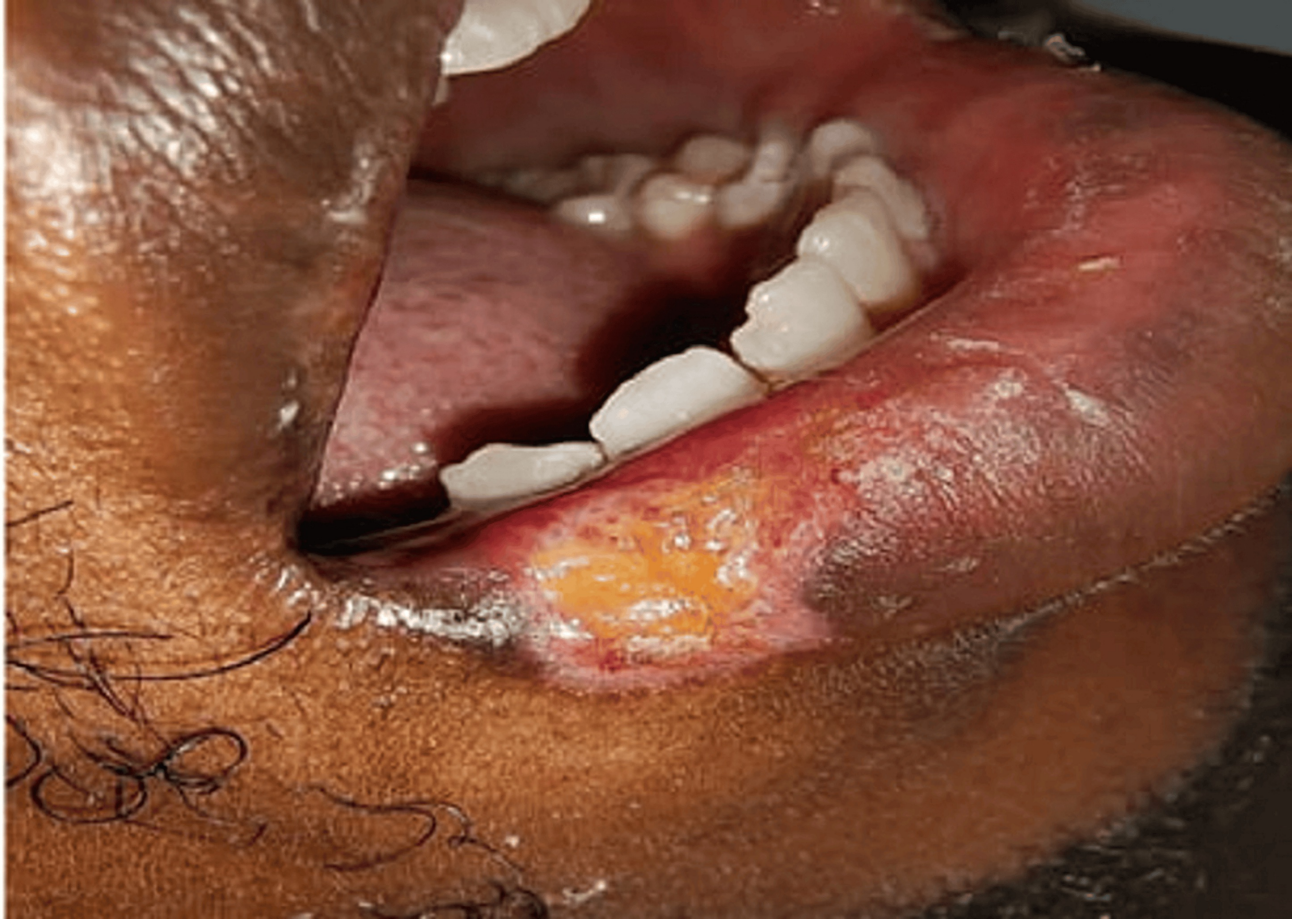
Initial visit with a nonhealing ulcer A nonhealing ulcer for five weeks due to iatrogenic trauma on the right lower lip

A 29-year-old man visited the Department of Oral Medicine and Radiology with a chief complaint of pain from an ulcer in the right lower lip for five weeks. The pain was intense and caused the patient difficulty in eating and speaking. The patient’s history revealed extraction of the third molar of the right lower posterior region five weeks previously, during which iatrogenic trauma occurred on the lip and persisted with no signs of healing. On examination, the ulcer was present on the right lower lip and was approximately 1.5 × 1 cm with irregular margins, sloping edges, tenderness, and a floor covered with yellowish slough (Figure 4).

Basic blood investigations were within normal limits. Thus, a provisional diagnosis of a chronic nonhealing traumatic ulcer was made. The patient received an explanation of the procedure, and consent was taken along with ethical approval for safety.

**Figure 5:**
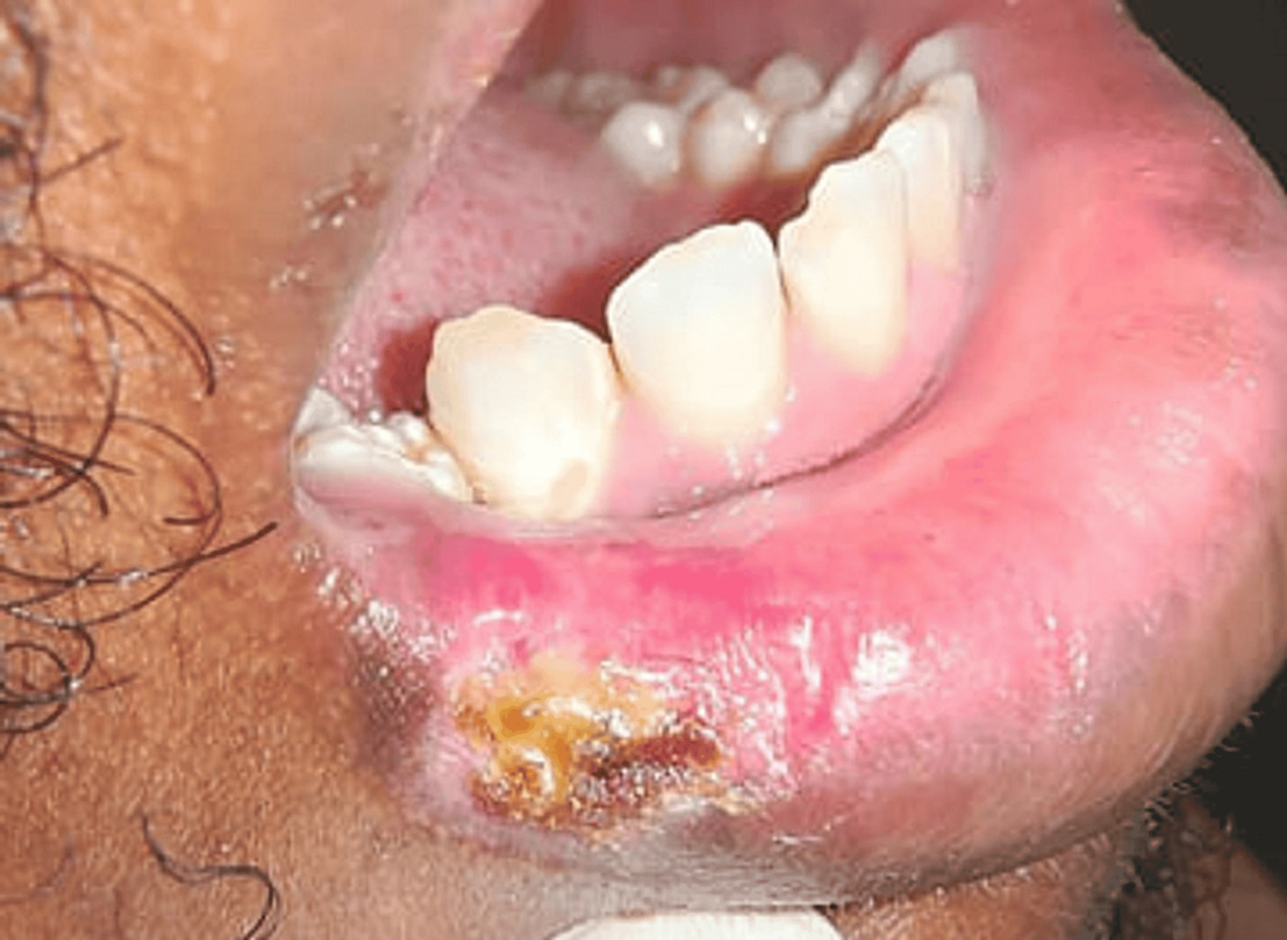
Day three with decreased size and healing ulcer with crust LLLT was given at 660 nm, 100 mW, and 6 J/Point. The patient showed 80% improvement at the third visit.

Initially, the ulcer was cleaned and irrigated with betadine gauze followed by LLLT with all safety measures at 660 nm, 100 mW, and 6 J. Contact was made for one minute at each of the four points on the ulcer every other day for 10 days. No drugs were used during the procedure. The patient’s pain was reduced by 40% after the first session and 80% after the third session (Figure 5).

**Figure 6:**
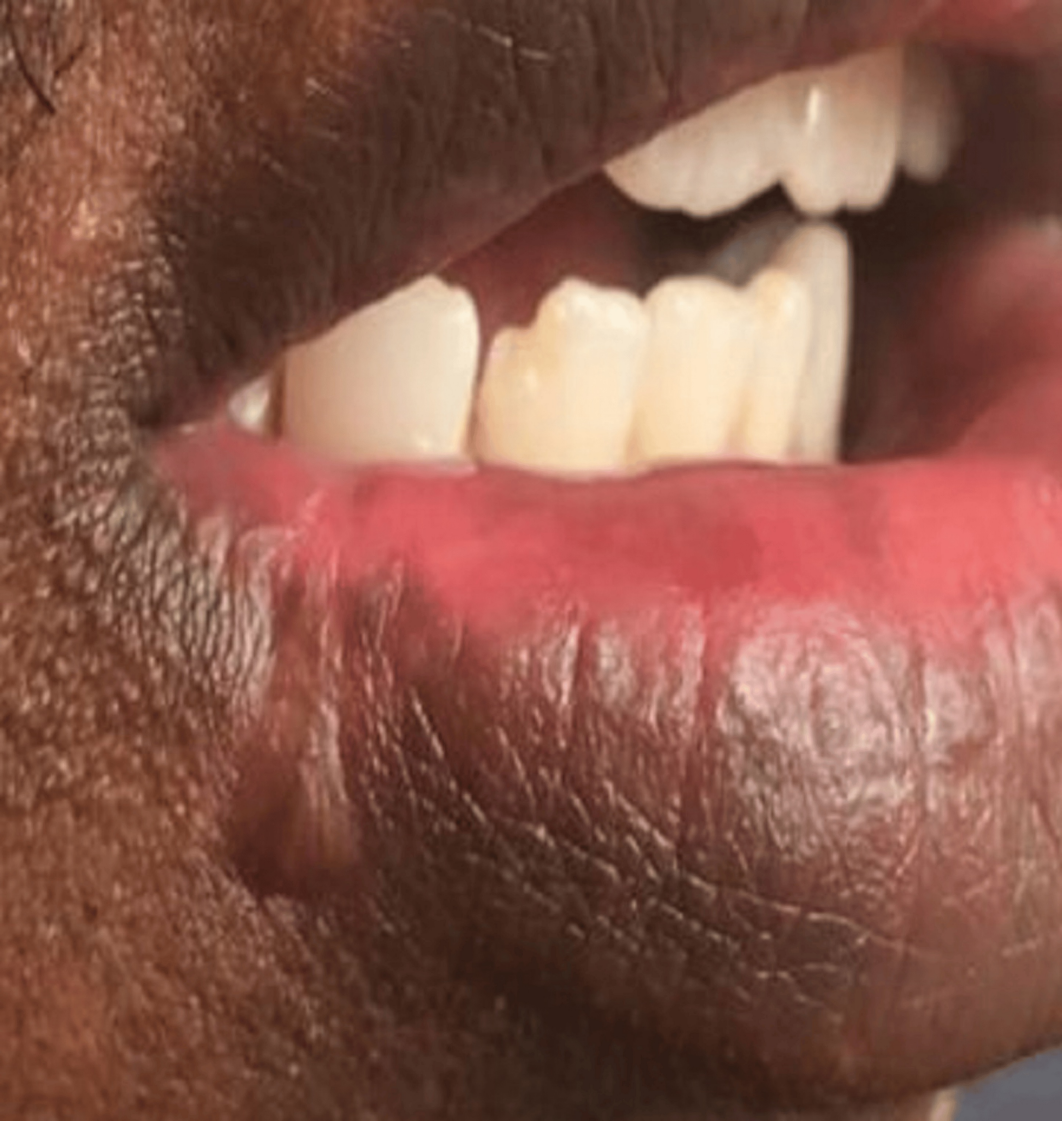
Complete healing at the fifth visit. LLLT was given at 660 nm, 100 mW, and 6 J/Point. The patient showed complete improvement at the fifth visit.

After the fifth visit, the patient’s pain decreased significantly, along with ulcer size and inflammation (Figure 6).

## Discussion

Conventional wound treatments face challenges due to the development of drug resistance and the potential for side effects. Insufficient oxygen supply in wounds, especially diabetic ulcers, hinders the natural healing process [1]. Alternative healing methods are needed to address these limitations [2,3].

Photobiomodulation (PBM) is known to accelerate wound healing, reduce inflammation, relieve pain, and help repair nerve damage [4,5]. Laser therapy, now called PBM, was first explored as a treatment for orofacial diseases in 2014 after Endre Mester described its biostimulation effect in 1967 [6,8].

PBM uses specific wavelengths of light (red and infrared) that penetrate the skin and stimulate healing by affecting cell components like cytochrome C oxidase and antennae pigments, which ultimately impact mitochondria [7]. This further helps in the production of ATP and the dissociation of nitric oxide. Increased ATP production by mitochondria stimulates fibroblasts, promotes collagen formation, and increases microcirculation, which helps accelerate wound healing [9].

The release of nitric oxide shifts cell redox potential toward greater oxidation and increased reactive oxygen species (ROS); this promotes transcriptional changes (such as κβ), which prevent apoptosis and cell death. Overall, this promotes growth factors and antioxidant responses and stimulates repair [10,11].

PBM relieves pain by blocking pain signals and reducing inflammatory chemicals. Its effectiveness depends on factors such as light wavelength, power, dose, duration, and application mode [12].

The main challenge in laser therapy clinics is defining the dose parameters. Rashidi et al. suggested that most guidelines show that energy density per treatment session should be within the range of 0.1-12.0 J/cm2 [10]. Suter et al.’s systemic review suggested different protocols for pain relief and wound healing by diode lasers as well as various non-ablative CO2 laser therapies of the neodymium-doped yttrium aluminum garnet laser [13]. Maya et al. showed the use of antimicrobial photodynamic therapy and PBM to treat palatal ulcers and heal necrotic lesion bacterial load [14-16].

## Conclusions

PBM therapy is a good alternative treatment option for chronic nonhealing and traumatic oral ulcers with the advantage of being a treatment that is drug-free, easy to use, and noninvasive. Two cases of chronic oral ulcers showed pain relief, faster healing, and better patient acceptance with PBM. Further studies are needed to compare PBM with conventional treatments.
